# Supplementary material for: Land-use change undermines the stability of avian functional diversity
Source: Nature. 2025 Nov 26;649(8096):381–7. doi: 10.1038/s41586-025-09788-0 (PMC12779574; doi:10.1038/s41586-025-09788-0)
Supplement: Supplementary file 1 — This file contains Supplementary Methods, Supplementary Discussion, Supplementary Tables 1–4, Supplementary Fig. 1 and Supplementary References. Supplementary Database 1 (hosted separately at https://zenodo.org) contains all the source data and code required to reproduce results and figures. The separate data files and code are available at Zenodo (https://zenodo.org/records/17184411). [file 41586_2025_9788_MOESM1_ESM.pdf]

nature portfolio
https://doi.org/10.1038/s41586-025-09788-0

Supplementary information

Land-use change undermines the stability of avian functional diversity

In the format provided by the authors and unedited

SUPPLEMENTARY INFORMATION

Land-use change undermines the stability of avian functional diversity

Thomas L. Weeks, Patrick A. Walkden, David P. Edwards, Alexander C. Lees, Alexander L. Pigot, Andy Purvis, Joseph A. Tobias

***Corresponding author:** j.tobias@imperial.ac.uk

Supplementary methods

Definitions and assumptions

(i) Resilience

Resilience is made up of two different components: resistance and recovery[1,2]. Resistance is defined as the capacity of a system to withstand disturbance and maintain its structure and function; recovery refers to the ability of the system to return to its original state following disturbance (Fig. 1). We are not able to investigate how functional diversity and redundancy contribute to ecosystem recovery as this would require time-series data. Instead, our study explicitly focuses on resistance as the first line of defence for an ecosystem facing disturbance and other anthropogenic threats.

(ii) Functional traits predict ecological processes

Our analyses assume that functional trait diversity present in species assemblages is linked to the delivery of ecological function and resilience[3–8]. In birds, the correlation between restricted sets of morphological traits and dietary niche is weak in some taxonomic groups[9,10]. However, wider sampling of morphological traits strongly predicts diet, food type, foraging behaviour, lifestyle and dispersal ability at a global scale[11–14]. This connection between morphological variation and niche complementarity across multiple dimensions of the ecological niche suggests that the diversity of traits within an assemblage should reflect the filling of functional roles in a local ecosystem.

A fundamental correlation between trait diversity and the rate and resilience of ecological processes is predicted by theory[15] and makes intuitive sense. However, the association is difficult to demonstrate empirically, particularly when dominant species disproportionately influence the measured function. Nonetheless, many studies support the view that FD and redundancy predict ecosystem multifunctionality and stability across a broad spectrum of contexts[16–18]. Plant FD has been shown to correlate with productivity, biomass, and carbon uptake[19,20], whereas animal FD can predict biogeochemical cycling[16], carrion removal[21], decomposition[22], and crop pollination[4,23]. Experiments have shown that animal trait redundancy boosts ecosystem multifunctionality by compensatory processes that buffer the effects of species loss[24]. Although evidence from birds is sparse, previous studies have shown a correlation between avian functional richness and rates of insect predation[25], pest control[26,27] and seed dispersal[28].

(iv) Demand for ecological functions after land-use change

Declining FD in anthropogenic landscapes may reflect the absence of ecological demand. According to this viewpoint, the loss of biodiversity and associated functions is not a problem because the need for ecological roles has been removed. Why do croplands or urban landscapes need frugivorous animals, for example, if the fruiting trees have gone? There are three main reasons. First, previous research has found that animals may be filtered from an environment long before their ecological function has expired. In birds, local extinction of frugivores[29–31] and nectarivores[32] often occurs prior to the eradication of their food-source, leaving an unmet demand for the ecological services they provide. Second, apart from the

most extreme cityscapes and agricultural monocultures, most anthropogenic environments continue to require ecological processes such as pollination, seed dispersal and pest control[33]. Third, landscapes with adequate functional diversity and redundancy are essential to maintain resilient ecosystems with the capacity for recovery and restoration. That is, maintaining a range of ecological roles and functions in an ecosystem means that it is primed for rapid recovery if conditions change or improve[34].

(iv) Species assemblages

The sampling unit in our analyses is the species assemblage, defined as an aggregation of organisms occurring in spatial proximity (i.e. not necessarily interacting) within a standard spatial and temporal extent defined by the biodiversity survey. Field survey data typically produces lists of co-occurring species but no evidence that they are interacting[35]. We therefore avoid the term 'community' as this implies interactions and interdependencies among species. Because the calculation of functional metrics and extinction curves is not possible when assemblages contain very few species, we group together survey data from different points within the same study block, time period and land-use class so that our study assemblages are sized appropriately for our analyses (see below).

Bird survey data

(i) Compiling surveys

We initially sampled all bird surveys from the PREDICTS database[36], some of which contained unsuitable data. We removed 38 datasets that lacked abundance data, or where sampling was not representative of the entire assemblage, for example through targeted sampling of subsets of species or sampling methods with severe biases. We removed camera trap surveys, which tend to report very few bird species, and retained intensive mist-netting surveys as these can provide a reasonable estimate of total bird diversity[37,38].

As a second stage, we conducted an intensive systematic literature review to identify studies fitting criteria predefined by PREDICTS[36]. We searched Web of Science using Boolean strings based on keywords: "(Birds OR avian) AND (biodiversity OR species diversity OR species richness OR species abundance OR community composition) AND ("land use") AND (old growth OR reserve* OR disturbed OR secondary OR logged OR logging OR fragment* OR remnant* OR plantation* OR silviculture OR agriculture OR cropland OR pasture OR grazing OR urban OR clear-cut* OR regeneration) AND (geographic terms)". Further studies were identified from reference lists. After sorting outputs by relevance, we contacted authors to request use of data. After contacting >200 authors, we received 29 suitable datasets. Finally, to improve sampling of extreme diversity gradients, we integrated data from intensive surveys in the Amazon[39] and Bornean rainforests[40]. The initial dataset contained 147 study landscapes, four of which are sampled using intensive mist-netting surveys.

(ii) Standardizing sampling units

Most datasets in our initial sample contained survey sites across a single geographical region and sampling was conducted over a restricted time-period. Other datasets contained survey

data from multiple distinct geographical regions or sampled the same area multiple times across different seasons. Some of these more complex datasets are restructured within PREDICTS so that survey sites are nested within temporal or geographical study-blocks, and study-blocks nested within overall study landscapes. However, 17 datasets did not follow this nested design, so – to ensure consistency – we restructured them into seven study landscapes with 2–5 study-blocks each. For additional surveys identified through our literature survey, we processed 16 unstructured datasets by assigning survey sites into study blocks, to break up survey data into portions with clear geographical or temporal demarcations. For each study block, we restricted data to a single season, so that we can account for annual and seasonal effects in our analyses (see below). One study contained two different point-count surveys differing only in the size of the sampling radius at each sampling location[41]. In this case, we discarded the study with the smallest point-count radius to reduce the risk of incomplete sampling. In total, we removed this pseudo-replicated dataset and condensed 106 datasets into 96 distinct study landscapes.

In PREDICTS, a single survey site can be defined in a variety of ways depending on sampling procedure and the rationale behind data collection. In studies treating single point-counts as communities, we aggregated clusters of such points within study blocks, forming small groups of survey sites characterised by the same land-use type and use intensity. This batching together of survey sites reduced the number of potential species assemblages but provided a better representation of overall diversity within assemblages, facilitating the calculation of functional metrics. One drawback of this process is that it increases variation in sampling effort between sites in some study landscapes. To account for this, we rescaled sampling effort by dividing the number of surveys for each site by the maximum sampling effort for a site in the same study landscape, following standard methods used in the PREDICTS dataset[36]. We then transformed abundance into effort-corrected abundance by dividing abundance measurements by the rescaled sampling effort.

To integrate additional survey data from independent studies in Amazonia[39] and Borneo[40], we transformed the data to fit the same nested structure described above and converted site-level descriptions of land-use type into categories consistent with definitions used in PREDICTS. Of particular importance were bird surveys conducted by the Sustainable Amazon Network (RAS), a comprehensive avian survey of Amazonian rainforest[39]. The RAS project sampled bird diversity over an area of ~72500km^2, consisting of 36 catchment areas surveyed by 4–12 line transects each (median = 10.5 transects). We treated each catchment area as a study block and each transect as a survey site. During RAS surveys, species presence was recorded at three points along each line-transect (0, 150 and 300m) and each transect was sampled multiple times by different observers (median 3; range 1–4). We estimated the relative abundance of species at each transect as the number of individuals reported divided by the sum of the number of observers at each sampling point along the transect. Borneo surveys[40] produced point-count data from 10 survey sites over ~300km^2, including primary forest sites and oil palm plantations. The structure of this dataset is similar to PREDICTS and readily incorporated into our dataset.

(iii) Classifying land-use

In alignment with most previous studies, we use the term land-use as short-hand for all human land-uses and natural (pristine) land-cover types which are arguably not used by humans. The land-use type of each assemblage was classified using descriptions extracted from each published survey. Classifications were made according to the predominant habitat, age class and use-intensity of the surveyed landscape. Initially we classified survey sites based on their six predominant habitat classes (primary vegetation, secondary vegetation, plantation forests, pasture, cropland, urban). We then split our primary vegetation and urban categories according to use-intensity. We classified minimal-use primary vegetation sites as pristine primary vegetation which was used as our proxy for undisturbed natural habitat. In this context, minimal-use habitat refers to intact natural vegetation, including survey sites with small-scale disturbance such as paths or occasional hunting. Although our "pristine primary" sites are probably not strictly pristine (100% natural state), we use this term as short-hand to discriminate from our 'disturbed primary" sites.

All other primary sites were either subjected to higher intensity disturbances (including selective logging, bushmeat extraction or clear-felling) or were found in contexts classified as urban or suburban. These sites were defined as disturbed primary vegetation. Based on previous analyses indicating greater impacts on avian functional trait diversity found in intensely urbanized areas[33], villages and extensively managed green spaces were defined as minimal-use urban sites while suburban and fully urban landscapes were grouped as intense-use urban habitat.

To account for the effects of vegetation structure at different successional stages[42,43], we split secondary vegetation into four age classes (mature, intermediate, young, indeterminate). As climax vegetation tends to be younger in the tropics[44], we classified tropical and non-tropical vegetation differently. Following Hudson et al.[36], mature vegetation was >30 years old in tropics and >75 years old in non-tropics; intermediate vegetation was 10–30 years old in tropics and 30–75 years old in non-tropics; and young vegetation was <10 years old in tropics and <30 years old in non-tropics. Indeterminate age secondary vegetation was removed from our analysis.

(iv) Survey methods and limitations

Data from different study landscapes vary widely for numerous reasons, including differences in sampling regime, survey techniques and the level of expertise in the field survey team. This means that bird diversity data cannot be directly compared across study landscapes. To overcome this problem, we add study landscape and study block as random intercepts in our models, following previous analyses[45–47]. This hierarchical approach effectively forces our model to make comparisons between study sites within the same study block, limiting our test to species assemblages sampled with the same methods. In other words, our global models aggregate data points within random effect groupings (study landscapes and study blocks), and then use a maximum likelihood method to select a single model estimate that best fits all the grouped data. This approach deals with many potential biases across different surveys by limiting comparisons to surveys undertaken by the same team, at the same season, and during the same period of the day.

The main weakness of this modelling approach is that it cannot address biases operating within study-blocks, such as variation in detection probability across land-use types. For example, our diversity indices are subject to sampling biases that may underestimate bird diversity in primary forests (the dominant primary habitat in our study). Tall, dense vegetation in primary forests makes birds less visible and harder to detect during surveys, particularly in the forest canopy[48]. It is therefore possible that widespread reports of high bird diversity and abundance in disturbed primary and secondary vegetation[49] at least partly reflect increased detectability in comparison with undisturbed primary vegetation. In particular, the lower, more discontinuous canopy of secondary forests, and the open aspect of most agricultural habitats, may lead to longer-range detections and more complete species sampling. Given the wide variety of survey methods across our sample, it is not possible to account for detection biases using methods such as occupancy models or distance sampling. However, we do not think that detection biases explain our results. On the contrary, imperfect detection probably leads to far greater underestimates of true bird diversity in primary forests than in any anthropogenic habitat. This means that our methods are likely to underestimate declines in FD and redundancy after land-use change and that any further correction for this bias would almost certainly increase support for our main conclusions.

(v) Taxonomic standardization

Bird survey data follows a variety of conflicting taxonomic treatments, both within PREDICTS and across all other sources used in this study. Avian taxonomy is in constant flux, with some treatments splitting widespread species into several geographically isolated daughter lineages[50]. To integrate datasets into a single species list, we converted all species-level taxa reported in surveys to BirdLife International taxonomy using a published crosswalk[51]. To minimise the risk of inaccurate taxonomic conversion and misidentifications, we assessed updated species classifications by checking whether survey data intersected with geographical range maps[52]. We found that coordinates of most species observations in our dataset (n = 106973; 88.7%) fell within the geographical range reported for each species. In these cases, we assumed the original species name used in published surveys reflects current species limits. When the survey observation coordinates fell outside the range polygon for a given species, we overlaid range polygons for all possible confusion lineages (that is, all daughter species split by BirdLife International from the relevant parent species). When only one daughter species range overlapped with the survey site (n = 4561; 3.7%), this species was selected for our analyses.

In a minority of cases, it was not possible to select a candidate species using range maps because survey localities overlapped with two or more daughter species ranges (n = 2261; 1.8%) or did not intersect with any range map of potential daughter species (n = 8132; 6.5%). In such cases, we manually selected the most likely species based on local abundance or proximity to localities reported in the eBird database (www.eBird.org), which provides precise observation locations including recent or extralimital records that extend beyond the boundaries of the BirdLife International range polygon[53].

To deal with a few cases (n = 751; 0.6%) where observations could not be assigned to a particular taxon, we created pseudo-species representing an average of all potential daughter species. In total, we used data from 73 pseudo-species present in 133 assemblages.

The inclusion of pseudo-species was preferred to removing taxa from the assemblage altogether because FD measures are sensitive to missing data[54]. Although the use of pseudo-species may introduce a degree of uncertainty, the scale of error is very low because phenotypic traits tend to be highly conserved in avian sister species, or larger sets of daughter species previously considered conspecific[51].

Our final dataset inludes a total of 3696 species units in 1281 bird assemblages sampled across 98 study landscapes in six continents (Supplementary Table 1).

Calculating functional diversity and redundancy

Functional trait diversity can be calculated as the total size of a convex hull volume encompassing the traits of all species in an assemblage[55]. However, standard methods based on species averages are insensitive to intraspecific variation and difficult to translate into redundancy. To provide a more nuanced and mechanistic link to functional redundancy, we calculated functional metrics using the Trait Probability Density (TPD) package in R[56]. The TPD method uses multivariate trait data sampled across a species assemblage to generate a multidimensional trait space, which is then subdivided into grid cells. Trait variation within a species can then be used to define which grid cells it is likely to occupy, representing the potential positions in which that species can maintain a population of individuals. Each species therefore has its own density kernel in trait space, representing a Hutchinsonian niche or n-dimensional hypervolume[57]. The advantage of this approach is that functional redundancy can be calculated as the amount of overlap between species in this hypervolume, since any two species sharing the same or overlapping positions in trait space are assumed to have overlapping ecological roles.

To estimate functional metrics, we begin by constructing TPDs for each study landscape. All individual density kernels for species detected in the landscape are merged into a single landscape-level TPD which is divided into equal-sized grid cells. All species kernels in this TPD are assigned a value reflecting the relative abundance of the species across the landscape[56]. Two outputs are then calculated for each grid-cell: (1) the number of species density kernels that overlap the grid cell (M); and (2) the sum of all density kernel values that overlap the grid cell (A).

Although TPDs are generated at the landscape-level, FD and redundancy are calculated at the assemblage-level using the *REND* and *redundancy* functions in the TPD package[56]. These functions generate smaller assemblage-level TPD subsets by extracting the relevant species from the local assemblage. For each assemblage, FD is calculated as the number of grid cells in which probability of occupancy by at least one species is >0. The functional redundancy of each grid cell is calculated as the product of M and A, from which the assemblage-level redundancy is calculated as the sum of grid cell redundancy values, minus 1. After subtracting 1, assemblage redundancy expresses the average number of species that could be removed from each grid-cell without reducing the total functional volume. FD is unrelated to species abundance, but the redundancy value for a particular grid cell is increased if the average relative abundance of species occupying that cell is high. Thus, species abundance influences functional redundancy.

An alternative approach is to estimate functional trait divergence as Rao's Quadratic Entropy (Q), then to calculate redundancy as either the difference between Rao's Q and the Simpson index[58], or the complement of "uniqueness", where uniqueness is calculated as Rao's Q divided by Simpson's diversity index[59]. However, Rao's Q has some limitations in the context of our study, particularly because it decreases with the addition of a generalist species occupying the centre of functional trait space[60]. We chose not to use Rao's Q as we do not find it logical, from the perspective of our hypotheses, that the addition of more generalist species should decrease ecosystem functionality[61].

(i) Estimating the position of species in functional trait space

The availability of species trait data for birds allows us to plot the position of each species in functional trait space, reflecting its likely ecological function. To achieve this using TPDs, we generated morphometric niche axes from eight morphological trait values grouped into locomotory (tail length, tarsus length, wing length) and trophic traits (beak width, beak depth, beak length [culmen], beak length [tip-to-nares]). We performed a two-step PCA on these traits to create three distinct niche axes representing variation in body size (PC1), along with the strongest locomotory trait and trophic trait axes independent of size (see Methods; Extended Data Fig. 2b). A fourth axis was the dispersal trait (Hand-wing Index [HWI]).

We processed these morphological niche axes alongside our dietary niche data to identify the likely position of each species in trait space. However, it is difficult to create TPDs with four or more dimensions because the total size of the TPDs increases exponentially with additional traits[62]. It is also non-trivial to convert proportional diet data into a continuous metric required to generate a TPD[63]. Therefore, we converted our data into a distance matrix, which we back-transformed into three-dimensional coordinates representing a species' position in multidimensional trait-space, as proposed by Laliberte & Legendre (2010)[64]. Using proportional diet data and derived trait-axes, we calculated a functional dissimilarity matrix with Gower distances using the package *gawdis* in R[65]. The *gawdis* function ensures that each of our five ecological trait axes contribute equally to the final dissimilarity matrix. To achieve this, the *gawdis* function alters the weight of each trait axes' contribution to multivariate dissimilarity until all univariate dissimilarity matrices have a similar correlation to the final distance matrix[65]. This means *gawdis* can handle proportional diet data by creating a combined dissimilarity matrix from all nine resource proportions and treating this as the univariate dissimilarity matrix for all species' diets. Once created, we back-transformed our multivariate dissimilarity matrix into coordinates in three-dimensional trait space using classical multidimensional scaling. This approach ensures that, for each landscape, the distance between species coordinates represents the distances between species represented in our multivariate dissimilarity matrix[64].

(ii) Accounting for intraspecific variation

In addition to the position of each species in trait space, the calculation of TPDs requires an estimate of intraspecific variation along each dimension. This estimate is used to calculate a kernel probability density (IV kernel) around each species coordinate, representing all possible positions of the species in the functional space. Landscape-level TPDs are then constructed by aggregating individual species probability density kernels. The species mean

trait value is located at the centre of these density kernels with the highest probability, and the extent of the kernels reflects intraspecific variation. The extent of functional overlap (redundancy) among species therefore depends on intraspecific variation in trait values.

The most accurate approach for estimating intraspecific trait variation requires direct measurements of large samples of individuals[62]. However, this is rarely feasible in macro-scale analyses because of a shortfall in trait data[66]. Birds, for example, have the most comprehensive individual-level trait data for any major taxonomic group, yet the availability of morphometric trait measurements varies widely across species[51]. Dietary data are even more patchy, and generally only available as species-level estimates[11,67].

To account for these biases, we estimated intraspecific trait variation using a plug-in kernel density bandwidth estimator implemented with the Hpi.diag function in the *ks* package[68]. This method – widely used in previous studies[55,61,68] – calculates a smoothed kernel around each species' mean trait value by selecting the bandwidth that produces the most accurate overall density surface across trait space. Specifically, the package selects the bandwidth that minimizes the Asymptotic Mean Integrated Squared Error (AMISE) – the expected estimation error of trait distributions. The main advantages of the bandwidth estimator are that it allows us to (1) estimate intraspecific trait variation for all species, including those with limited sampling of individual-level trait measurements, and (2) integrate species-level data such as dietary proportions into estimates of intraspecific variation. The bandwidth estimator varies according to the distances between species-mean positions in trait space which can differ between assemblages. Therefore, to ensure we estimated a species' intraspecific variation consistently, we ran the bandwidth estimator on each species assemblage and took a landscape-level average for each dimension (that is, we averaged across all species assemblages in each study landscape).

The approach we use to estimate intraspecific variation allows us to calculate more realistic redundancy values compared to methods which consider all pairwise distances between species in the same assemblage[59]. In these methods, redundancy can fluctuate based on raw distances between species in trait space regardless of whether they perform similar ecological roles. Our use of probability density kernels ensures that redundancy is only affected by the species with high enough trait similarity to share the same region of trait space[62].

(iii) Role of species richness in assemblage function

Assemblages with higher species richness (SR) often have higher FD because additional species increase the chance of providing a unique function. The positive relationship between SR and FD can cause difficulties when comparing between assemblages surveyed with varying sampling efforts, or between geographically distant assemblages with inherently different levels of SR (e.g. tropical vs temperate comparisons). A widespread method to account for systematic differences in SR involves calculating observed FD scores and then standardizing them by subtracting the FD values expected for an equal-sized assemblage with a random species composition[69–71]. However, we do not use this method in our study because comparing standardized FD of assemblages masks important effects of SR on ecosystem function.

To assess the impact of land-use change on functional metrics, we followed previous analyses using mixed effects models to compare metrics between multiple studies with variable sampling methods and distinct regional species pools[45,72,73]. Specifically, we include study landscape and study block as random intercepts to ensure that functional metrics are directly compared among nearby assemblages sampled in the same season. Therefore, our analyses retain the effects of SR on assemblage function while controlling for most temporal and spatial biases.

(iv) Assessing the resilience of key ecological processes

To examine effects of land-use change on ecological stability, we recreated TPDs for trophic guilds (see Methods) and recalculated functional richness and redundancy scores using the same methods described above. As variation in diet is relatively constrained within trophic guilds, we excluded dietary proportion data and instead calculated guild-specific TPDs using only morphological data. We focused on four functional groups with adequate samples across each of our land-use types: generalists (n = 1271 assemblages), granivores (n = 1271 assemblages), frugivores (n = 944 assemblages) and invertivores (n = 1274 assemblages). Sample sizes vary because some survey sites do not contain representative species from the respective dietary guilds, or too few representatives to calculate TPDs.

Functional stability and resilience

Estimates of FD and functional redundancy only provide limited insight into the stability and resilience of ecosystems. Global-scale trait datasets for birds[51] allow us to examine these questions further using comprehensive sets of response and effect traits to quantify how functional vulnerability and resistance vary across land-use types. We scaled all sensitivity scores by their standard deviation within each assemblage.

(i) Accounting for species sensitivity

General sensitivity

We calculated sensitivity scores for each species under two different species-loss scenarios: trait-based and rarity-based. Our main trait-based sensitivity scores were based on four general response traits that influence sensitivity to a broad spectrum of environmental disturbances (Fig. 3; Table S1). Larger-bodied birds more frequently undergo declines or extinction in human-modified and fragmented habitats[74,75]. Studies have also shown that land-use change and habitat fragmentation have greater negative impacts on dietary specialists[76] and species with poor dispersal ability[13]. Finally, geographical range size correlates with conservation status and small-ranged bird species are less likely to persist in human-modified landscapes[75,77,78].

We estimated relative body size by extracting the size-related PC from the 2-step PCA described above. We obtained geographical range sizes from AVONET[51] and converted them to inverse (negative) log-transformed range sizes so that higher values represent smaller ranges (Fig. 1). This aids interpretation in our analyses because species with restricted distributions are more sensitive to environmental change. Using data from Pigot et al.[11], we calculated dietary specialism for each species as the proportion of the diet represented by

their primary food source, such that higher values indicate greater specialisation. Finally, we used inverse (negative) log-transformed Hand-wing Index (nHWI) as a measure of dispersal limitation[13]. Within each study landscape, we scaled the response trait values by their standard deviation and calculated trait-based sensitivity scores for each species as the mean value across these four traits. By scaling sensitivity scores within each study landscape, we approximate the sensitivity scores relative to the entire regional species pool rather than the species observed in the assemblage.

Climate sensitivity

The strength of general indicators is that they are relevant to multiple disturbance types, reflecting the fact that the source and severity of future disturbance is unknown. However, previous studies indicate that trait selection can substantially influence the sensitivity values we assign to each species. To evaluate resilience in the context of a more explicit threat, and to assess whether our results are robust to the choice of response traits, we recalculated functional resistance using extinction curves generated with a set of response traits specifically related climate change[79] (see Table S2 for rationale).

Dispersal strongly influences the ability of species to respond to climate change[80,81], so we kept dispersal limitation as one of our key response traits. From other published datasets, we extracted generation length estimates[82] and minimum elevation[83], because high . Finally, we extracted average temperature seasonality from the CHELSA bioclim dataset v2.1[84]. The CHELSA dataset contains global estimates of local intra-annual temperature variation equal to the standard deviation of monthly mean temperatures across each year using global data from 1981–2010 within grid cells at 30 arcsecond resolution. To extract an average seasonality estimate for each species, we overlaid climate data with expert-drawn distributional ranges provided by Birdlife International (2021)[52]. Grid cells that fell within each species' distribution range were averaged using a Behrmann equal area projection, disregarding cells with less than 50% overlap. As high temperature seasonality is related to climatic tolerance[79], we took the inverse values (i.e. negative) values of the average temperature seasonality across the range (i.e. Climate limitation). Following methods used to generate our general sensitivity scores, we scaled all traits by their standard deviation and took an average as our climate change sensitivity scores.

Rarity

As an alternative to trait-based methods, we used a different approach based on the concept that the rarest species in a community are the most sensitive to future perturbations. We calculated rarity-based sensitivity scores as the inverse of abundance for each species in the assemblage. This approach assumes that the likelihood of local extinctions will be inversely correlated to the number of individuals surviving in the landscape, as smaller populations are more sensitive to population bottlenecks and stochastic events driving fluctuations in abundance[85,86].

(ii) Calculating functional vulnerability

When species are removed from an assemblage, redundancy buffers against functional declines via compensatory dynamics mediated by other species able to perform similar

roles[24]. Therefore, it follows that the removal of a species with a unique set of traits will result in larger functional losses than the removal of a redundant species. In other words, the positive effects of assemblage-level redundancy on resistance depend on whether the most likely species to be removed from an assemblage are unique or redundant. To explore this question, we calculated a novel metric which we refer to as functional vulneraility (FV). FV is an assemblage-level metric which calculates the covariance between how sensitive a species is (i.e. how likely it is to be removed by environmental change) and how much redundancy the species provides to the assemblage.

To calculate FV, we first estimated the redundancy value for each species. We then generated assemblage-level TPDs by aggregating the individual species density kernels of all species present. Next, for each species we constructed a pseudo-assemblage – defined as the same assemblage with the focal species removed. We then used the *redundancy* function in the TPD package[56] to calculate functional redundancy for the full assemblage and each individual pseudo-assemblage. We estimated the amount of functional redundancy attributed to each species in the assemblage (i.e. species redundancy) as the difference between the full assemblage redundancy and the redundancy of the respective pseudo-assemblage. Finally, we calculated the Spearman's rank correlation coefficient between species redundancy scores and species sensitivity scores (trait-based and rarity-based). A high covariance indicates that the most sensitive species are also the species with the highest functional redundancy in the assemblage. We took the inverse (negative) of these covariance values as our trait-based and rarity-based FV values, respectively. This is more intuitive and helps interpretation because we are mainly interested in how the distribution of redundancy (with respect to sensitivity) affects the vulnerability of assemblage function.

(iii) Calculating functional resistance

Generating extinction curves

We calculated our resistance metrics by tracking the decline in FD as species are removed from the assemblage to generate extinction curves, following previous methods[62,87–89]. We generated three sets of extinction curves per assemblage by sequentially removing species according to each of our sensitivity scores (general sensitivity, climate sensitivity and rarity). After each species removal, we recalculated FD to generate extinction curves describing how the ecological functioning of each assemblage declines as species losses accumulate. Species in the same assemblage can have the same trait- and rarity-based sensitivity scores if they share a similar combination of response traits or are equally abundant. Therefore, we repeated our analyses 100 times and randomized the order in which we removed species with similar sensitivity scores with each iteration. Thus, for each assemblage, we generated 3 sets of 100 extinction curves, one for each sensitivity score.

Smaller assemblages may lack redundancy because they contain few species well dispersed in trait space. In these cases, a single species accounts for a higher proportion of total FD than a single species in an assemblage with higher species richness. Thus, when a species is removed from a small assemblage there is likely to be steeper declines in FD. To account for this effect of starting species richness, we standardized our extinction curves by both initial species richness and initial FD by expressing both species richness and FD as proportions of their starting value. Using this technique, removing a species from a smaller

assemblage will lead to a larger decline in FD but will also lead to a greater proportional loss of species compared to the initial species richness. Therefore, the extinction curve should differ little between a species-poor assemblage and a species-rich assemblage, provided the amount and distribution of redundancy per species is the same across both assemblages.

Area under the extinction curve

For each assemblage, we generated three separate functional resistance values for each of our assemblages based on our three sets of extinction curves (general sensitivity, climate sensitivity & rarity). For each set of extinction curves, we calculated the area under the extinction curve (AUC) value and took the mean across all 100 values as our final functional resistance estimates (Supplementary Fig. 1). Alternative estimates of functional resistance can be generated by creating virtual communities with altered abundance and redundancy structures[90]. This method allows a calculation of resistance through the comparison of the observed extinction curve to theoretical best- and worst-case scenarios. We do not adopt this approach because our analysis compares the extinction curves of observed communities to those in pristine primary vegetation, removing the need to normalize our data against theoretical baselines.

Functional diversity half-life

One drawback of AUC-based calculations of functional resistance is their sensitivity to an early loss of function. If one of the first species removed provides large amounts of unique function, this can lead to a low AUC value overall, even if FD remains stable thereafter. To assess the robustness of our AUC method, we use an alternative approach based on the half-life of the extinction curve ($t_{1/2}$), which is less sensitive to the sequence in which function is lost[91]. We defined $t_{1/2}$ as the proportion of species that need to be removed for FD to decline by 50% (Supplementary Fig. 1).

We ran two univariate mixed effects models to evaluate the impacts of land-use change on functional resistance ($t_{1/2}$) of each assemblage under our two species-loss scenarios (general trait-based and rarity-based). We kept the structure of our models similar to our main AUC analysis. We included study landscape and study-block as random effects and land-use was split into four categories: i) primary vegetation, ii) secondary vegetation, iii) agriculture (including plantation forests), and iv) urban. Models were interpreted by comparing the change in estimated effect size for each land-use type using primary vegetation as our reference category. Similar to our main AUC analysis, $t_{1/2}$ declined in all disturbed land-use types, particularly in urban and agricultural landscapes (Extended Data Fig. 7).

Simulations using passive (probability-weighted) extinction

Our main AUC and $t_{1/2}$ analyses are based on simulated extinction curves generated by sequential removal of species in order of their sensitivity, with a single species removed at each timestep. This method is designed to estimate the effects of land-use change on functional resistance assuming widespread species losses projected across a range of biomes and ecosystems[92]. However, it could be argued that our analyses do not account for differences in species sensitivity between land-use types. In particular, the assumption that bird species will continue to undergo local extinction at a constant rate with increasing

disturbance may be invalid because vulnerable species have been filtered from disturbed landscapes, leaving highly tolerant species that may survive indefinitely[93,94]. Therefore, it is possible that assemblages in disturbed habitats will lose species at a slower rate than more natural landscapes, which could reduce the rate of FD declines in response to further threats.

To account for the reduced sensitivity of species in anthropogenic landscapes, we devised a probabilistic extinction procedure in which species were not forced to go extinct at each timestep. We assigned an extinction probability to every species in our sample (n = 3696) based on their general sensitivity scores. Thus, species with small range sizes, large body sizes, poor dispersal abilities and specialised diets have a high probability of being removed at each timestep while species insensitive to general threats are less likely to be removed. We set the absolute probability of extinction to vary between p = 0.66 for the most sensitive species and p = 0.33 for the least sensitive species in our sample. We assign a non-zero extinction probability to species associated with farmland and urban habitats because there is a long history of such species being driven extinct by agricultural intensification and increasing urbanisation. Even previously abundant garden birds can undergo severe declines and local extinctions through unexpected threats such as disease[95] and pollutants[96].

To generate functional resistance estimates using extinction probabilities, we re-ran extinction simulations over 100 timesteps, with the likelihood of each species going extinct at each timestep determined by its extinction probability score. A key aspect of this analysis is that assemblages with a high proportion of tolerant species do not necessarily lose species at each timestep. We set a minimum of zero and a maximum cap of 2 extinctions at each time-step to reflect natural extinction scenarios playing out over an extended period rather than large numbers of species disappearing concurrently[97]. In cases where >2 species had been assigned for removal, we removed the two species with the highest sensitivity scores. This allowed tolerant species to remain in the assemblage for longer periods, generating longer-tailed extinction curves. Based on these curves, we calculated AUC as a metric of functional resistance. Unlike our main analyses, we did not standardise AUC by assemblage SR.

The results were similar to the results of our main analyses, showing lower levels of functional resistance in human-modified landscapes (Extended Data Fig. 7). In other words, the increased tolerance of species in human-modified habitats appears to be insufficient to buffer assemblages against declines in functional resistance driven by losses of functional redundancy.

Since we do not standardize by SR, the number of timesteps required for the extinction curve to reach 0 is increased in assemblages with higher SR, generating higher resistance values. We think this is reasonable because, in real ecosystems, the number of species present should have a direct impact on the functional resistance of an assemblage because it reflects the number of species available to perform certain functions. Nonetheless, without standardizing our extinction curves by SR, it is harder to determine whether resistance is driven by greater SR or whether increased niche overlap generates more stable assemblages. To examine whether estimated declines in resistance were simply a reflection of starting assemblage SR, we calculated relative redundancy (that is, the average niche overlap per species) within the assemblage using in-built functions from the TPD package in R[56]. Next, we ran a linear mixed-effect model to assess how functional resistance was affected by relative redundancy in the assemblage. We ran this model across all three trait-based AUC

extinction simulation scenarios (general, climate and passive) to allow comparison. We found significant positive relationships between relative redundancy and all three of our functional resistance metrics (Extended Data Fig. 8). As expected, the relationship between relative redundancy and functional resistance was weakest in our passive extinction scenario. This is likely because the resistance values under our passive extinction scenario are more affected by both starting species richness and the presence or absence of highly tolerant species with low sensitivity scores.

Robustness and sensitivity analyses

(i) Alternative measure of intraspecific variation

The scale of redundancy and functional richness depends on estimates of intraspecific trait variation used to define the overall extent of each individual species density kernel used in TPDs. To assess whether our results are robust to different approaches for estimating intraspecific variation, we repeated our analysis using direct measures of intraspecific variation. We achieve this using individual-level morphological data published in the AVONET database, which contains measurements of morphological traits taken from museum specimens and live captures, sampled across multiple individuals of almost all bird species[51]. Measurements of live birds differ fractionally from measurements taken from dried museum specimens, but the differences are so slight[51] we used both forms of measurement to maximize individual-level sampling. In total, our sample contained 45488 trait measurements from 18183 individual birds, representing all species in our study.

Repeating the two-step PCA approach described above, we used the same eight morphological trait values to generate three distinct niche axes representing variation in body size, locomotory and trophic traits (see Methods; Extended Data Fig. 2b). However, as the number of individuals measured for each species in AVONET varies widely, we cannot perform dimensionality reduction through PCAs that use individual level data because this would bias derived trait values towards patterns in species with higher numbers of individual measurements. To address this problem, we performed each PCA on species mean trait values and then used the rotation algorithm to predict values for each individual measured (henceforth: Individual-level PCA). Similarly, we constructed the dispersal niche axis by scaling individual-level HWI measurements with the standard deviation of the species-level HWI values.

To align with three-dimensional TPDs in our main analysis, we further reduced the dimensionality of this new set of TPDs by performing a final individual-level PCA. That is, we performed a PCA on species mean values and used the rotation algorithm to predict individual-level values across each of our three derived trait axes (locomotory, trophic, dispersal). To run the individual-level PCA we first we calculated the mean trait value across each trait axis and quantified intraspecific variation across each axis as the standard deviation of all individual measurements. For a minority (8.5%) of study species, it was not possible to run individual-level PCAs because measurements were only available for <4 individuals in the AVONET database[51]. For these species, we estimated their position in trait space using their species mean trait values and inferred kernel density bandwidth estimates using the

average intraspecific trait variation within the assemblage. We then used this data to construct new landscape-level TPDs using the *TPDsMean* function from the *TPD* package in R[56].

Based on these TPDs, we recalculated our three main assemblage measures (FD, functional redundancy and FV). We then analysed whether land-use change affected these metrics using the same mixed effects modelling approach as we used for our main analysis. The results derived using this method of estimating intraspecific variation similar to our main analyses (Extended Data Fig. 4).

(ii) Assessing the role of trophic generalists

We explored the effects of land-use change on the stability of specific ecosystem functions provided by birds by analysing how functional resistance responded to land-use change within specific dietary guilds. We extracted estimates of species diets as proportions across nine major resource types[11] and assigned species into dietary groupings based on their primary food source, provided they consumed greater than 60% of their diet from a single resource type. We focused on three well-surveyed dietary guilds with wide spatial coverage: invertivores, frugivores and granivores. Results from our analyses indicated that declines in functional resistance are strongest in invertivores and frugivores on the ability of bird assemblages to maintain key ecosystem services provided by these guilds. However, as omnivores can contribute to the same ecosystem processes provided by specialists, it is possible that increases in the functional resistance of omnivorous species can partially account for the declines in function within specialist clades.

To assess whether the contribution of omnivores to resource consumption across different food sources influenced our conclusions, we relaxed the criteria for assigning dietary guilds to allow any species that consumed >25% of a single food source to be allocated into that dietary guild. This relaxed criterion increased the samples for each dietary guild: granivores (n = 1271 assemblages), frugivores (n = 1271 assemblages), invertivores (n = 1274 assemblages). Using this approach, we allowed omnivorous species to occur in multiple dietary groups, representing their contribution to multiple ecosystem processes. After generating these dietary groups, we repeated our trait-based extinction simulations to generate new extinction curves for each assemblage, taking the AUC as our measure of functional resistance. As expected, inclusion of more generalists into our diet groupings resulted in more modest declines in functional redundancy in disturbed habitats (Extended Data Fig. 6), reflecting the higher resistance of generalist species (Fig. 3c). Nonetheless, frugivores and invertivores continue to sharply decline in functional redundancy (Extended Data Fig. 6), indicating that generalist species do not compensate for functional losses associated with the local eradication of specialist species.

(iii) Checking and accounting for spatial autocorrelation

Our models are hierarchical in structure, making it difficult to incorporate covariance structures that account for spatial autocorrelation between local survey sites in the same study landscape into our global models. Therefore, following Newbold et al.[45], we assessed the extent of spatial autocorrelation in the residuals of our land-use change models. We grouped our data by study landscape and ran four linear models using lme4 package in R[98] across each landscape (n = 98). Each model assessed whether land-use change affected the FD, functional

redundancy, FV or functional resistance of assemblages in the study landscape. We then assessed the level of spatial autocorrelation in the model using Moran's I test from the *spdep* package in R[99]. As spatial autocorrelation may occur at the study-block level, we repeated our analysis by running our four models over each study-block in our data (n = 375). It is not possible to calculate Moran's I on study landscapes or study-blocks that have less than three assemblages, removing approximately half of our dataset from the sensitivity analysis.

We found that spatial autocorrelation in data from study landscapes and study blocks was very low (estimated at ~4% in the study-block analysis. To assess whether spatial autocorrelation affected our results, we repeated our main analyses (i) excluding all study landscapes with a significant Moran's I test and (ii) further excluding all study landscapes for which we were unable to calculate a Moran's I. The results in all cases continued to support our conclusions (Extended Data Fig. 9).

Supplementary discussion

Estimating variation in functional diversity and redundancy

(i) Land-use types

The largest declines in FD were detected in urbanized areas, where land-use change is perhaps most extreme, corroborating the patterns described by Sol et al.[33]. This effect is probably driven by the loss of species maladapted to urbanized landscapes, including ecological specialists located at the extremities of functional trait space[100,101]. We show that losses of FD are also accompanied by severe declines in functional redundancy, indicating that urban assemblages are dominated by only a few, typically generalist species. The ecological functions provided by urban bird assemblages are supplied by a limited number of species, leaving some bird-mediated processes under-represented and susceptible to additional environmental pressures. Across other land-use types reductions in FD and redundancy are more moderate.

Although agricultural landscapes show similar patterns to urban settings, the impacts of land-use change are less severe, particularly outside the tropics. Irrespective of age class, assemblages in secondary vegetation sites are expected to have lower FD and assemblage-level redundancy (Fig. 2b-d). However, FD declines are marginal in mature secondary regrowth and redundancy is only significantly different to pristine-primary vegetation in the youngest of our secondary vegetation classes. This pattern supports previous assessments which indicate that, when allowed to regenerate, secondary forests can maintain high levels of avian phylogenetic and functional diversity[102,103].

These findings are expected because the key components of variation in secondary vegetation – canopy complexity, stand density and resource availability – are heavily time-dependent and increase with the duration of regrowth according to the successional stage of the forest[104]. Therefore, older types of secondary vegetation have more complex vegetation structure capable of maintaining a wider variety of ecological functions[105]. Nonetheless, our analyses reveal that the FD of species assemblages in mature secondary forests remain depleted in frugivore and invertivore guilds, and redundancy is lower throughout the assemblage (Extended Data Figs. 5 & 6). Thus, even the oldest undisturbed secondary forests may fail to recapture the full variety and stability of specific ecosystem services provided by unique species, which may influence assemblage composition across multiple trophic levels and alter ecosystem function and productivity[106,107].

Our analyses rely on space-for-time substitutions which may ignore temporal dynamics in assemblage composition, particularly in the case of lag-times to local colonization and extinction after land-use change[108]. For example, space-for-time approaches may underestimate FD and redundancy in secondary forests if species continue to colonize late-successional vegetation over long periods of time. Conversely, assemblages in recently cleared or modified landscapes may represent a lag phase before extinction debts have been fully paid[109], in which case predicted changes in both FD and redundancy may underestimate further losses of ecosystem function over time. To account for these temporal dynamics, time-series data are needed to quantify how FD and redundancy change over time after disturbance events, ideally based on repeated surveys over several decades at particular sites.

(ii) Trophic groups

In our dataset, declines in FD and redundancy are primarily driven by losses of frugivores and invertivores (Extended Data Figs. 5 & 6). These comprise the largest and perhaps most ecologically valuable components of bird diversity. Frugivore diversity promotes seed dispersal, maintaining the structure and composition of forest plant communities[30,110], particularly in the tropics where up to 70-94% of woody species depend on animals for seed dispersal[111]. Similarly, invertivores play key roles by regulating insect populations, including many herbivorous insects, agricultural pests and disease vectors[112]. Previous work has shown that avian invertivores are key to maintaining healthy photosynthesis and plant growth[113,114], reducing leaf damage and improving agricultural yield[115]. Similar declines in frugivore and invertivore FD after land-use change have been identified at local scales[76,116–118], suggesting that these guilds have elevated sensitivity to human pressure[119], either because they are susceptible to reductions in habitat availability and food supply[120,121], or – in the case of tropical invertivores – because dispersal constraints cause population declines in fragmented or open habitats[13].

(iii) Latitude

The effects of land-use change on FD and redundancy appear to vary with latitude. In temperate and polar regions, both FD ($\hat{\beta}$: -0.077, $P = 0.425$) and functional redundancy ($\hat{\beta}$: -0.188, $P = 0.067$) underwent slight, statistically non-significant declines in disturbed primary vegetation (Extended Data Fig. 10). This contrasts with tropical assemblages, where the disturbance of primary vegetation leads to a significant increase in both FD ($\hat{\beta}$: 0.361, $P = 0.008$) and redundancy ($\hat{\beta}$: -0.239, $P = 0.024$). This increase in diversity from primary to disturbed tropical forests may be partly explained by survey biases because birds tend to be easier to detect in less-dense vegetation where they are more visible in the forest canopy or in flight[48]. Alternatively, bird species richness and abundance may peak in disturbed tropical forests[49]. These increases are associated with gains in frugivore and invertivore diversity (Extended Data Figs. 5 & 6), consistent with some Amazonian studies[122]. In general, forest disturbance can boost populations of some avian guilds through the proliferation of palatable leaf-growth at forest gaps and edges, and the consequent increased availability of insect prey, as well as increased flowering and fruiting rates associated with disturbed primary forests[48,49,123].

On average, species assemblages have higher redundancy in the tropics (Extended Data Fig. 10). However, tropical assemblages also undergo sharp declines in FD and redundancy when land-use change progresses beyond disturbed primary vegetation, especially in agricultural and urbanized landscapes (Extended Data Fig. 10). Outside the tropics, the difference between FD of primary vegetation and human-modified assemblages is reduced. This geographical variation in the impacts of land-use change on functional attributes may be driven by three main factors. First, species at higher latitudes are relatively disturbance tolerant[94], potentially due to historically high levels of landscape disturbance which filters out species sensitive to environmental change[93,124,125]. Second, many tropical species are highly sedentary habitat specialists with poor dispersal or gap-crossing abilities[12,126,127], reducing their fitness in fragmented habitats[13,128]. Third, tropical

assemblages contain many species with specialised diets (such as obligate frugivores[129]) or restricted range sizes[130], both attributes strongly associated with increased sensitivity to land-use change[47,70,76]. Thus, despite high levels of redundancy in tropical assemblages, land-use change may have a disproportionately larger impact on the types of species typically found in these regions driving widespread losses of function.

One noteworthy observation from our models is that plantation forests appear to show a reversed pattern, with non-tropical plantation forests undergoing substantially larger declines in FD and redundancy than observed in tropical plantation forests. This may relate to differences in forestry practices. For example, plantation forests at higher latitudes are typically monocultures of exotic conifers or eucalyptus, with very low species richness and colonisation rates[131,132]. By contrast, tropical plantation forests are often broad-leaved trees associated with humid forests (e.g. rubber, *Hevea brasiliensis*; teak, *Tectona grandis*) with much higher species richness, particularly when situated adjacent to source populations in primary or secondary tropical forests[133,134].

Moving beyond birds as a study system

Birds offer an ideal system for global studies of functional stability because they are well-surveyed and widely used as indicators of ecosystem health[135], with the most comprehensive trait datasets currently available[51]. Nonetheless, the impacts of land-use change on birds may not map directly onto other taxonomic groups or trophic levels. Further studies are needed across different systems to assess the generality of our results. This will require intensive surveying of a wider selection of plant and animal groups, as the relevant information remains taxonomically patchy or geographically restricted[136,137]. In addition, further sampling of relevant traits is an urgent priority for most clades, to move beyond coarse categorical life-history traits and trait imputation methods[138,139]. Some progress has been made towards this goal with recent advances in species-trait databases[51,140–142] although substantial data gaps remain[143]. The completion of ecological trait data sets at global scales will allow a more nuanced assessment of the functional implications of land-use change for entire ecosystems[7].

Supplementary tables

Table S1: Geographical distribution and sources of avian land-use change datasets. Assemblage numbers per continent are provided below, with study landscapes in brackets. PREDICTS A: after excluding inappropriate samples (see Methods), 64 study landscapes were included from the latest published release of the PREDICTS database[36]. PREDICTS B: we conducted an intensive literature review to improve sampling, adding a further 27 study landscapes, all of which which have now been integrated into the latest release of PREDICTS database[144]. PREDICTS C: additional studies independently uploaded to PREDICTS[144]. Independent: additional study landscapes from the Amazon[39] and Bornean rainforests[40]. All data has been reorganized into a standardized format for the purposes of our analyses (see Methods).

Continent	PREDICTS A	PREDICTS B	PREDICTS C	Independent
Africa	135 (14)	20 (6)	24 (2)	0 (0)
Asia	150 (11)	47 (7)	3 (1)	10 (1)
Europe	58 (7)	229 (6)	0 (0)	0 (0)
North America	68 (6)	187 (5)	0 (0)	0 (0)
Oceania	93 (8)	0 (0)	4 (1)	0 (0)
South America	73 (18)	15 (3)	12 (1)	153 (1)
**Total**	**577 (64)**	**498 (27)**	**43 (5)**	**163 (2)**

Table S2: Rationale for selection of functional response traits. First column lists seven response traits used to generate species-level sensitivity scores (with data source in brackets). We selected response traits based on evidence linking variation in the trait to population declines or local extinctions in response to different disturbance scenarios: general threats and climate change. Traits with patchy sampling or requiring imputation were excluded.

Trait	Description	Rationale	Disturbance scenario	Results	References
Body size	Generated from locomotory and trophic trait PC1 (Table S1; Extended Data Fig. 2)	Larger-bodied species are less persistent in fragmented habitats; Population declines in human-modified habitats are more prevalent in large-bodied species; Predictor of IUCN threat level.	General	Fig. 3; Extended Data Figs. 7, 8 & 9	Gaston & Blackburn[145]; Keinath et al.[146]
Range size[52]	Total area of breeding and non-breeding geographical range	Persistent indicator of conservation status; Narrow ranged species more sensitive to land-use change; Species with restricted ranges disproportionately likely to undergo local extinctions.	General	Fig. 3; Extended Data Figs. 7, 8 & 9	Newbold et al.[47]; Staude et al.[147]
Dietary specialism[11]	Proportion of diet obtained from the most used food-source across nine major resource types	Reduced diversity of dietary specialists in human-modified landscapes; Population sizes more sensitive to loss of habitat compared to generalist species; Specialised diets more sensitive to land-use intensification.	General	Fig. 3; Extended Data Figs. 7, 8 & 9	Boyles & Storm[148]; Kellner et al.[149]
Dispersal limitation[51]	Inverse (i.e. negative) hand-wing index	Reduced capacity to cross habitat-gaps leading to high sensitivity to habitat fragmentation and land-use intensification; Reduced capacity to adapt to climate change; Population declines more prevalent in sedentary species compared to migratory species.	General; Climate change	Fig. 3; Extended Data Figs. 7, 8 & 9	Claramunt et al.[150]; Weeks et al.[13]; Rochat et al.[151]

Table S2 (continued)

Trait	Description	Rationale	Disturbance scenario	Results	References
Generation rate[82]	Mathematical approximation of generation rate from age of first breeding, longevity and adult survival rate.	Poor adaptability to environmental change; Slower recovery rates in response to disturbances; Disproportionately influenced by climate-change.	Climate Change	Extended Data Figs. 7 & 8	Pacifici et al.[79]; Etard & Newbold[152]; Capdevila et al.[1]; Maron et al.[153]
Temperature seasonality[84]	Mean temperature seasonality across the species breeding and non-breeding range.	Species persisting in more stable climates are more impacted by climate change; Limited thermal tolerance increases exposure to land-use changes.	Climate Change	Extended Data Figs. 7 & 8	Pacifici et al.[79]; Williams et al.[154]
Minimum elevation[83]	Lowest limit of the elevational range	Species restricted to higher elevations have narrower climatic ranges and are less able to move upslope in response to climate change.	Climate Change	Extended Data Figs. 7 & 8	Freeman et al.[155]; Pacifici et al.[79]; Urban et al.[156]; White & Bennett[157]

Table S3: Rationale for selection of functional effect traits. Table shows the five derived niche axes used to generate trait probability density hypervolumes. Constituent traits for trophic, locomotory and dispersal axes are extracted from AVONET[51]. Trophic and locomotory axes are generated from a principal component analysis of their constituent traits. PC2 represents variation in traits, independent of body size.The size axis is derived from the first principal component (PC1) of the trophic and locomotory PCAs (Extended Data Fig. 2). Alongside each axis we document the ecological characteristics infered from the derived trait axis with references for the inferences.

Functional effect trait axes	Constituent traits	Ecological inference	References
Trophic axis	Beak length (culmen), Beak length (tip-to-nares), Beak width, Beak depth	Diet; feeding action (e.g. probing, cracking, hammering); food type	Felice et al.[10]; Sayol et al.[14]
Locomotory axis	Tail length, Tarsus length, Wing length	Foraging niche; foraging strategy (e.g. walking, sallying, gleaning)	Fitzpatrick[155]; Einoder & Richardson[158]; Miles & Ricklefs[159]; Miles et al.[160]
Size axis	Trophic PC1, Locomotory PC1	Energy requirements; total consumption of resources; foraging niche	Hudson et al.[161]; Steuer et al.[162]
Dispersal axis	Hand-wing index	Seed dispersal; foraging distance; nutrient transfer	Sheard et al.[12]; Weeks et al.[163]; Gonzalez-Varo et al.[164]
Dietary axis[11]	Proportional diet consumption across nine major resources	Likelihood of species interactions within and between trophic levels	Pigot et al.[11]

Table S4: Phylopic. Image credits and licenses for silhouettes used in figures.

Taxa	Credit	License	Figure
Campephilus magellanicus	Edwin Price	CC0 1.0	Fig. 3a
Chionis minor	Alexandre Vong	CC0 1.0	Fig. 3a
Geranium maculatum	Mason McNair	CC0 1.0	Fig. 3a
Malus pumila	T. Michael Keesey	PDM 1.0	Fig. 3a
Ploceidae	Lucy the bob man	PDM 1.0	Fig. 3a
Popillia japonica	Andy Wilson	CC0 1.0	Fig. 3a
Riparia riparia	Bruno Maggia	CC0 1.0	Fig. 3a
Xenicus gilviventris	Ferran Sayol	CC0 1.0	Fig. 3a
Ramphastos	Edwin Price	CC BY 4.0	Fig. 3a
Cariama cristata	George Edward Lodge & T. Michael Keesey	PDM 1.0	Extended Data Fig. 2

Supplementary figures

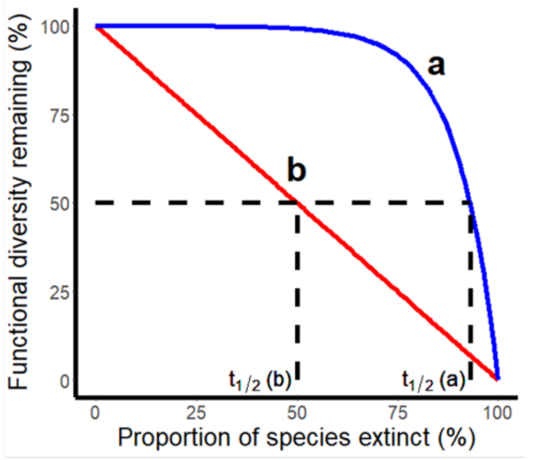

	a	b
**AUC**	8992.81	5000
$\mathbf{t_{1/2}}$	92.52	50

Supplementary Fig. 1: Theoretical representation of extinction curves.

Coloured lines show two alternative types of extinction curves created by tracking the decline in functional diversity (FD; measured as functional richness) caused by the sequential removal of species from an assemblage. Blue extinction curve (**a**) depicts an assemblage with strong functional resistance (able to maintain relatively high FD despite increasing species loss). Red extinction curve (**b**) depicts an assemblage with weak functional resistance (FD declining linearly with species loss). We estimate functional resistance of the assemblage in two ways: the area under the curve (AUC), and the proportion of species remaining after the FD remaining is reduced to 50% (half-life; $t_{1/2}$).

Supplementary references

1. Capdevila, P. *et al.* Reconciling resilience across ecological systems, species and subdisciplines. *J. Ecol.* **109**, 3102–3113 (2021).
2. Hodgson, D., McDonald, J. L. & Hosken, D. J. What do you mean, 'resilient'? *Trends Ecol. Evol.* **30**, 503–506 (2015).
3. Cadotte, M. W., Carscadden, K. & Mirotchnick, N. Beyond species: Functional diversity and the maintenance of ecological processes and services. *J Appl. Ecol.* **48**, 1079–1087 (2011).
4. Greenop, A. Patterns of invertebrate functional diversity highlight the vulnerability of ecosystem services over a 45-year period. *Curr. Biol.* **31**, 4627-4634 3 (2021).
5. Miedema Brown, L. & Anand, M. Plant functional traits as measures of ecosystem service provision. *Ecosphere* **13**, 3930 (2022).
6. Tobias, J. A. A bird in the hand: Global-scale morphological trait datasets open new frontiers of ecology, evolution and ecosystem science. *Ecol. Lett.* **25**, 573–580 (2022).
7. Schleuning, M., García, D. & Tobias, J. A. Animal functional traits: Towards a trait-based ecology for whole ecosystems. *Funct. Ecol.* **37**, 4–12 (2023).
8. Miller-ter Kuile, A. *et al.* Functionalizing ecological integrity: using functional ecology to monitor animal communities. *Front. Ecol. Env.* **23***,* e2852, (2025).
9. Bright, J. A., Marugán-Lobón, J., Cobb, S. N. & Rayfield, E. J. The shapes of bird beaks are highly controlled by nondietary factors. *Proc. Natl. Acad. Sci. USA* **113**, 5352–5357 (2016).
10. Felice, R. N., Tobias, J. A., Pigot, A. L. & Goswami, A. Dietary niche and the evolution of cranial morphology in birds. *Proc. R. Soc. B* **286**, 20182677 (2019).
11. Pigot, A. L. *et al.* Macroevolutionary convergence connects morphological form to ecological function in birds. *Nat. Ecol. Evol.* **4**, 230–239 (2020).
12. Sheard, C. *et al.* Ecological drivers of global gradients in avian dispersal inferred from wing morphology. *Nat. Commun.* **11**, 2463 (2020).
13. Weeks, T. L. *et al.* Climate-driven variation in dispersal ability predicts responses to forest fragmentation in birds. *Nat. Ecol. Evol.* **7**, 1079–1091 (2023).
14. Sayol, F., Reijenga, B., Tobias, J. A. & Pigot, A. L. Ecophysical constraints on avian adaptation and diversification. *Curr. Biol.* **35**, 1326–1336 (2025).
15. Ceulemans, R. The effects of functional diversity on biomass production, variability, and resilience of ecosystem functions in a tritrophic system. *Sci. Rep.* **9**, 7541 (2019).

16. Gagic, V. Functional identity and diversity of animals predict ecosystem functioning better than species-based indices. *Proc. R. Soc. B* **282**, 20142620 (2015).
17. Huang, X. Functional diversity drives ecosystem multifunctionality in a Pinus yunnanensis natural secondary forest. *Sci. Rep.* **9**, 6979 (2019).
18. Lefcheck, J. S. & Duffy, J. E. Multitrophic functional diversity predicts ecosystem functioning in experimental assemblages of estuarine consumers. *Ecology* **96**, 2973–2983 (2015).
19. Hao, M. Functional traits influence biomass and productivity through multiple mechanisms in a temperate secondary forest. *Eur. J. For. Res.* **139**, 959–968 (2020).
20. Wu, Y. *et al.* Functional diversity explains ecosystem carbon storage in subtropical forests. *Glob. Change Biol.* **31**, 70120 (2025).
21. Mosman, J. D. *et al.* Scavenger richness and functional diversity modify carrion consumption in the surf zone of ocean beaches. *ICES J. Mar. Sci.* **80**, 2024–2035 (2023).
22. Dekanová, V., Novikmec, M., Svitková, I. & Svitok, M. Functional diversity of shredders, not species richness, drives the decomposition rate of leaf litter in ponds. *Front. Ecol. Evol.* **11**, 1286672 (2023).
23. Woodcock, B. A. *et al.* Meta-analysis reveals that pollinator functional diversity and abundance enhance crop pollination and yield. *Nat. Commun.* **10**, 1481 (2019).
24. Yeeles, P., Lach, L. & Hobbs, R. J. Functional redundancy compensates for decline of dominant ant species. *Nat. Ecol. Evol.* **9**, 779–788 (2025).
25. Barbaro, L. Bird functional diversity enhances insectivory at forest edges: a transcontinental experiment. *Divers. Distrib.* **20**, 149–159 (2014).
26. Barbaro, L. Avian pest control in vineyards is driven by interactions between bird functional diversity and landscape heterogeneity. *J. Appl. Ecol.* **54**, 500–508 (2017).
27. Martínez-Salinas, A. *et al.* Bird functional diversity supports pest control services in a Costa Rican coffee farm. *Agric. Ecosyst. Environ.* **235**, 277–288 (2016).
28. Saavedra, F. *et al.* Functional importance of avian seed dispersers changes in response to human-induced forest edges in tropical seed-dispersal networks. *Oecologia* **176**, 837–848 (2014).
29. Galetti, M. *et al.* Functional extinction of birds drives rapid evolutionary changes in seed size. *Science* **340**, 1086–1090 (2013).
30. Terborgh, J. *et al.* Tree recruitment in an empty forest. *Ecology* **89**, 1757–1768 (2008).

31. Sethi, P. I. A. & Howe, H. F. Recruitment of hornbill-dispersed trees in hunted and logged forests of the Indian Eastern Himalaya. *Conserv. Biol.* **23**, 710–718 (2009).
32. Anderson, S. H., Kelly, D., Ladley, J. J., Molloy, S. & Terry, J. Cascading effects of bird functional extinction reduce pollination and plant density. *Science* **331**, 1068–1071 (2011).
33. Sol, D. *et al.* The worldwide impact of urbanisation on avian functional diversity. *Ecol. Lett.* **23**, 962–972 (2020).
34. Hatfield, J. H., Banks-Leite, C., Barlow, J., Lees, A. C. & Tobias, J. A. Constraints on avian seed dispersal reduce potential for resilience in degraded tropical forests. *Funct. Ecol.* **38**, 315–326 (2024).
35. Blanchet, F. G., Cazelles, K. & Gravel, D. Co-occurrence is not evidence of ecological interactions. *Ecol. Lett.* **23**, 1050–1063 (2020).
36. Hudson, L. N. *et al.* The database of the PREDICTS (Projecting Responses of Ecological Diversity In Changing Terrestrial Systems) project. *Ecol. Evol.* **7**, 145–188 (2017).
37. Martin, T. E. *et al.* Variability in the effectiveness of two ornithological survey methods between tropical forest ecosystems. *PLoS One* **12**, 0169786 (2017).
38. Mestre, L. A., Cochrane, M. A. & Barlow, J. Long-term changes in bird communities after wildfires in the central Brazilian Amazon. *Biotropica* **45**, 480–488 (2013).
39. Nunes, C. A. *et al.* Linking land-use and land-cover transitions to their ecological impact in the Amazon. *Proc. Natl. Acad. Sci. USA* **119**, 2202310119 (2022).
40. Edwards, F. A. *et al.* Does logging and forest conversion to oil palm agriculture alter functional diversity in a biodiversity hotspot? *Anim. Conserv.* **17**, 163–173 (2014).
41. Hvenegaard, G. T. Validating bird diversity indicators on farmland in east-central Alberta, Canada. *Ecol. Indic.* **11**, 741–744 (2011).
42. Drapeau, P. *et al.* Landscape-scale disturbances and changes in bird communities of boreal mixed-wood forests. *Ecol. Monogr.* **70**, 423–444 (2000).
43. Zhang, Q., Han, R. & Zou, F. Effects of artificial afforestation and successional stage on a lowland forest bird community in southern China. *Ecol. Manag.* **261**, 1738–1749 (2011).
44. Brown, S. & Lugo, A. E. The storage and production of organic matter in tropical forests and their role in the global carbon cycle. *Biotropica* **14**, 161–187 (1982).
45. Newbold, T. *et al.* Global effects of land use on local terrestrial biodiversity. *Nature* **520**, 45–50 (2015).

46. Newbold, T. *et al.* Global effects of land use on biodiversity differ among functional groups. *Funct. Ecol.* **34**, 684–693 (2020).
47. Newbold, T. *et al.* Widespread winners and narrow-ranged losers: Land use homogenizes biodiversity in local assemblages worldwide. *PLoS Biol.* **16**, 2006841 (2018).
48. Mayhew, R. J., Tobias, J. A., Bunnefeld, L. & Dent, D. H. Connectivity with primary forest determines the value of secondary tropical forests for bird conservation. *Biotropica* **51**, 219–233 (2019).
49. Malhi, Y. *et al.* Logged tropical forests have amplified and diverse ecosystem energetics. *Nature* **612**, 707–713 (2022).
50. Garnett, S. T. & Christidis, L. Taxonomy anarchy hampers conservation. *Nature* **546**, 25–27 (2017).
51. Tobias, J. A. *et al.* AVONET: morphological, ecological and geographical data for all birds. *Ecol. Lett.* **25**, 581–597 (2022).
52. Birdlife International. BirdLife Data Zone.
53. Sullivan, B. L. *et al.* eBird: A citizen-based bird observation network in the biological sciences. *Biol. Conserv.* **142**, 2282–2292 (2009).
54. Pakeman, R. J. Functional trait metrics are sensitive to the completeness of the species' trait data? *Meth. Ecol. Evol.* **5**, 9–15 (2014).
55. Villéger, S., Mason, N. W. H. & Mouillot, D. New multidimensional functional diversity indices for a multifaceted framework in functional ecology. *Ecology* **89**, 2290–2301 (2008).
56. Carmona, C. P., Bello, F., Mason, N. W. H. & Lepš, J. Trait probability density (TPD): measuring functional diversity across scales based on TPD with R. *Ecology* **100,** 02876 (2019).
57. Blonder, B. *et al.* New approaches for delineating n-dimensional hypervolumes. *Meth. Ecol. Evol.* **9**, 305–319 (2018).
58. Maure, L. A. *et al.* Functional redundancy in bird community decreases with riparian forest width reduction. *Ecol. Evol.* **8**, 10395–10408 (2018).
59. Ricotta, C. *et al.* Measuring the functional redundancy of biological communities: a quantitative guide. *Meth. Ecol. Evol.* **7**, 1386–1395 (2016).
60. Petchey, O. L. & Gaston, K. J. Functional diversity: back to basics and looking forward. *Ecol. Lett.* **9**, 741–758 (2006).

61. Sollow, A. R. & Polasky, S. Measuring biological diversity. *Env. Ecol. Stat.* **1**, 95–103 (1994).
62. Carmona, C. P., Bello, F., Mason, N. W. H. & Lepš, J. Traits without borders: integrating functional diversity across scales. *Trends Ecol. Evol.* **31**, 382–394 (2016).
63. Shanmugam, R. Applied compositional data analysis: with worked examples in R. *J Stat Comput. Simul.* **89**, 3165 (2019).
64. Laliberte, E. & Legendre, P. A distance-based framework for measuring functional diversity from multiple traits. *Ecology* **91**, 299–305 (2010).
65. De Bello, F., Botta-Dukát, Z., Lepš, J. & Fibich, P. Towards a more balanced combination of multiple traits when computing functional differences between species. *Meth. Ecol. Evol.* **12**, 443–448 (2021).
66. De Bello, F. *et al.* Raunkiæran shortfalls: challenges and perspectives in trait-based ecology. *Ecol. Mono.* **95**, 70018 (2025).
67. Wilman, H. *et al.* EltonTraits 1.0: Species-level foraging attributes of the world's birds and mammals. *Ecology* **95**, 2027-2027 (2014).
68. Duong, T. Ks: Kernel density estimation and kernel discriminant analysis for multivariate data in R. *J. Stat. Softw.* **21**, 1–16 (2007).
69. Bonfim, F. C. G., Dodonov, P. & Cazetta, E. Landscape composition is the major driver of the taxonomic and functional diversity of tropical frugivorous birds. *Landsc. Ecol.* **36**, 2535–2547 (2021).
70. Bregman, T. P. *et al.* Using avian functional traits to assess the impact of land-cover change on ecosystem processes linked to resilience in tropical forests. *Proc. R. Soc. B* **283**, 20161289 (2016).
71. Lee, M. B. & Martin, J. A. Avian species and functional diversity in agricultural landscapes: does landscape heterogeneity matter? *PLoS One* **12**, 0170540 (2017).
72. De Palma, A. *et al.* Dimensions of biodiversity loss: Spatial mismatch in land-use impacts on species, functional and phylogenetic diversity of European bees. *Divers. Distrib.* **23**, 1435–1446 (2017).
73. Etard, A., Pigot, A. L. & Newbold, T. Intensive human land uses negatively affect vertebrate functional diversity. *Ecol. Lett.* **25**, 330–343 (2022).
74. Allen, D. C. *et al.* Long-term effects of land-use change on bird communities depend on spatial scale and land-use type. *Ecosphere* **10**, 02952 (2019).
75. Barbaro, L. & Van Halder, I. Linking bird, carabid beetle and butterfly life-history traits to habitat fragmentation in mosaic landscapes. *Ecography* **32**, 321–333 (2009).

76. Newbold, T. *et al.* Ecological traits affect the response of tropical forest bird species to land-use intensity. *Proc. R. Soc. B* **280**, 20122131 (2013).
77. Harris, G. & Pimm, S. L. Range size and extinction risk in forest birds. *Conserv. Biol.* **22**, 163–171 (2008).
78. Phalan, B., Onial, M., Balmford, A. & Green, R. E. Reconciling food production and biodiversity conservation: Land sharing and land sparing compared. *Science* **333**, 1289–1291 (2011).
79. Pacifici, M., Visconti, P. & Butchart, S. Species' traits influenced their response to recent climate change. *Nat. Clim. Change* **7**, 205–208 (2017).
80. Boeye, J., Travis, J. M., Stoks, R. & Bonte, D. More rapid climate change promotes evolutionary rescue through selection for increased dispersal distance. *Evol. Appl.* **6**, 353–364 (2013).
81. Schloss, C. A., Nuñez, T. A. & Lawler, J. J. Dispersal will limit ability of mammals to track climate change in the Western Hemisphere. *Proc. Natl. Acad. Sci. USA* **109**, 8606–8611 (2012).
82. Bird, J. P. *et al.* Generation lengths of the world's birds and their implications for extinction risk. *Conserv. Biol.* **34**, 1252–1261 (2020).
83. Yang, J., Yang, C., Lin, H. W., Lees, A. C. & Tobias, J. A. Elevational constraints on flight efficiency shape global gradients in avian wing morphology. *Curr. Biol.* **35**, 1890–1900 (2025).
84. Karger, D. N. *et al.* Climatologies at high resolution for the earth's land surface areas. *Sci. Dat.* **4**, 1–20 (2017).
85. Lande, R. Risks of population extinction from demographic and environmental stochasticity and random catastrophes. *Am. Nat.* **142**, 911–927 (1993).
86. Curtis, J. R., Robinson, W. D., Rompré, G., Moore, R. P. & McCune, B. Erosion of tropical bird diversity over a century is influenced by abundance, diet and subtle climatic tolerances. *Sci. Rep.* **11**, 10045 (2021).
87. Galland, T., Carmona, C. P., Götzenberger, L., Valencia, E. & Bello, F. Are redundancy indices redundant? An evaluation based on parameterized simulations. *Ecol. Indic.* **116**, 106488 (2020).
88. Leitão, R. P. *et al.* Rare species contribute disproportionately to the functional structure of species assemblages. *Proc. R. Soc. B* **283**, 20160084 (2016).
89. Sasaki, T. *et al.* Differential responses and mechanisms of productivity following experimental species loss scenarios. *Oecologia* **183**, 785–795 (2017).

90. Auber, A. *et al.* A functional vulnerability framework for biodiversity conservation. *Nat. Commun.* **13**, 4774 (2022).
91. Fonseca, C. R. & Ganade, G. Species functional redundancy, random extinctions and the stability of ecosystems. *J. Ecol.* **89**, 118–125 (2001).
92. Leclère, D. *et al.* Bending the curve of terrestrial biodiversity needs an integrated strategy. *Nature* **585**, 551–556 (2020).
93. Balmford, A. Extinction filters and current resilience: the significance of past selection pressures for conservation biology. *Trends Ecol. Evol.* **11**, 193–196 (1996).
94. Betts, M. G. *et al.* Extinction filters mediate the global effects of habitat fragmentation on animals. *Science* **366**, 1236–1239 (2019).
95. Hanmer, H. J. *et al.* Habitat-use influences severe disease-mediated population declines in two of the most common garden bird species in Great Britain. *Sci. Rep.* **12**, 15055 (2022).
96. Peach, W. J., Vincent, K. E., Fowler, J. A. & Grice, P. V. Reproductive success of house sparrows along an urban gradient. *Anim. Conserv.* **11**, 493–503 (2008).
97. Ferraz, G. *et al.* Rates of species loss from Amazonian forest fragments. *Proc. Natl. Acad. Sci. USA* **100**, 14069–14073 (2003).
98. Bates, D., Mächler, M., Bolker, B. & Walker, S. Fitting linear mixed-effects models using lme4. *J. Stat. Softw.* **67**, 1–48 (2015).
99. Bivand, R. R packages for analyzing spatial data: a comparative case study with areal data. *Geogr. Anal.* **54**, 488–518 (2022).
100. Sorte, F. A. *et al.* The phylogenetic and functional diversity of regional breeding bird assemblages is reduced and constricted through urbanization. *Divers. Distrib.* **24**, 928–938 (2018).
101. Oliveira Hagen, E., Hagen, O., Ibáñez-Álamo, J. D., Petchey, O. L. & Evans, K. L. Impacts of urban areas and their characteristics on avian functional diversity. *Front. Ecol. Evol.* **5**, 84 (2017).
102. Edwards, D. P., Massam, M. R., Haugaasen, T. & Gilroy, J. J. Tropical secondary forest regeneration conserves high levels of avian phylogenetic diversity. *Biol. Conserv.* **209**, 432–439 (2017).
103. Hughes, E. C., Edwards, D. P., Sayer, C. A., Martin, P. A. & Thomas, G. H. The effects of tropical secondary forest regeneration on avian phylogenetic diversity. *J. Appl. Ecol.* **57**, 1351–1362 (2020).

104. Guariguata, M. R. & Ostertag, R. Neotropical secondary forest succession: changes in structural and functional characteristics. *Ecol. Manag.* **148**, 185–206 (2001).
105. Acevedo-Charry, O. & Aide, T. M. Recovery of amphibian, reptile, bird and mammal diversity during secondary forest succession in the tropics. *Oikos* **128**, 1065–1078 (2019).
106. Howe, F. & Smallwood, J. Ecology of seed dispersal. *Ann. Rev. Ecol. Syst.* **13**, 201–228 (1982).
107. Jactel, H. *et al.* The influences of forest stand management on biotic and abiotic risks of damage. *Ann. Sci.* **66**, 701 (2009).
108. De Palma, A. *et al.* Challenges with inferring how land-use affects terrestrial biodiversity: study design, time, space and synthesis. *Adv. Ecol. Res.* **58**, 163–199 (2018).
109. Kuussaari, M. *et al.* Extinction debt: A challenge for biodiversity conservation. *Trends Ecol. Evol.* **24**, 564–571 (2009).
110. Wotton, D. M. & Kelly, D. Frugivore loss limits recruitment of large-seeded trees. *Proc. R. Soc. B* **278**, 3345–3354 (2011).
111. Jordano, P. Fruits and frugivory. In *Seeds: the Ecology of Regeneration in Plant Communities*, pp. 18–61 (CABI, Wallingford UK, 2014).
112. Şekercioğlu, C. H. Increasing awareness of avian ecological function. *Trends Ecol. Evol.* **21**, 464–471 (2006).
113. Mooney, K. A. *et al.* Interactions among predators and the cascading effects of vertebrate insectivores on arthropod communities and plants. *Proc. Natl. Acad. Sci. USA* **107**, 7335–7340 (2010).
114. Van Bael, S. A., Brawn, J. D. & Robinson, S. K. Birds defend trees from herbivores in a Neotropical forest canopy. *Proc. Natl. Acad. Sci. USA* **100**, 8304–8307 (2003).
115. Philpott, S. M. *et al.* Functional richness and ecosystem services: Bird predation on arthropods in tropical agroecosystems. *Ecol. Appl.* **19**, 1858–1867 (2009).
116. Luck, G. W., Carter, A. & Smallbone, L. Changes in bird functional diversity across multiple land uses: Interpretations of functional redundancy depend on functional group identity. *PloS ONE* **8**, 63671 (2013).
117. Pineda-Diez De Bonilla, E., León-Cortés, J. L. & Rangel-Salazar, J. L. Diversity of bird feeding guilds in relation to habitat heterogeneity and land-use cover in a human-modified landscape in southern Mexico. *J. Trop. Ecol.* **28**, 369–376 (2012).

118. Shahabuddin, G., Goswami, R., Krishnadas, M. & Menon, T. Decline in forest bird species and guilds due to land use change in the Western Himalaya. *Glob. Ecol. Conserv.* **25**, 01447 (2021).
119. Lee, T. M. & Jetz, W. Unravelling the structure of species extinction risk for predictive conservation science. *Proc. R. Soc. B* **278**, 1329–1338 (2011).
120. Cleary, D. F. R. *et al.* Bird species and traits associated with logged and unlogged forest in Borneo. *Ecol. Appl.* **17**, 1184–1197 (2007).
121. Vetter, D., Hansbauer, M. M., Végvári, Z. & Storch, I. Predictors of forest fragmentation sensitivity in Neotropical vertebrates: A quantitative review. *Ecography* **34**, 1–8 (2011).
122. Barlow, J. & Peres, C. A. Avifaunal responses to single and recurrent wildfires in Amazonian forests. *Ecol. Appl.* **14**, 1358–1373 (2004).
123. Wunderle, J. M., Willig, M. R. & Henriques, L. M. P. Avian distribution in treefall gaps and understorey of *terra firme* forest in the lowland Amazon. *Ibis* **147**, 109–129 (2005).
124. Gámez-Virués, S. *et al.* Landscape simplification filters species traits and drives biotic homogenization. *Nat. Commun.* **6**, 8568 (2015).
125. Le Provost, G. *et al.* Land-use history impacts functional diversity across multiple trophic groups. *Proc. Natl. Acad. Sci. USA* **117**, 1573–1579 (2020).
126. Salisbury, C., Seddon, N., Cooney, C. & Tobias, J. A. The latitudinal gradient in dispersal constraints: ecological specialisation drives diversification in tropical birds. *Ecol. Lett.* **15**, 847–855 (2012).
127. Tobias, J. A. *et al.* Territoriality, social bonds, and the evolution of communal signaling in birds. *Front. Ecol. Evol.* **4**, 74 (2016).
128. Martin, A. E., Desrochers, A. & Fahrig, L. Homogenization of dispersal ability across bird species in response to landscape change. *Oikos* **126**, 996–1003 (2017).
129. Kissling, W. D., Böhning-Gaese, K. & Jetz, W. The global distribution of frugivory in birds. *Glob. Ecol. Biogeogr.* **18**, 150–162 (2009).
130. Belmaker, J., Şekercioğlu, C. H. & Jetz, W. Global patterns of specialization and coexistence in bird assemblages. *J. Biogeogr.* **39**, 193–203 (2012).
131. Goded, S. *et al.* Effects of eucalyptus plantations on avian and herb species richness and composition in North-West Spain. *Glob. Ecol. Conserv.* **19**, 00690 (2019).
132. Lindenmayer, D. *et al.* Richness is not all: how changes in avian functional diversity reflect major landscape modification caused by pine plantations. *Divers. Distrib.* **21**, 836–847 (2015).

133. Barlow, J. *et al.* Quantifying the biodiversity value of tropical primary, secondary, and plantation forests. *Proc. Natl. Acad. Sci. USA* **104**, 18555–18560 (2007).

134. Gardner, T. A., Barlow, J., Parry, L. W. & Peres, C. A. Predicting the uncertain future of tropical forest species in a data vacuum. *Biotropica* **39**, 25–30 (2007).

135. Fraixedas, S. *et al.* A state-of-the-art review on birds as indicators of biodiversity: Advances, challenges, and future directions. *Ecol. Indic.* **118**, 106728 (2020).

136. Hortal, J. *et al.* Seven shortfalls that beset large-scale knowledge of biodiversity. *Annu. Rev. Ecol. Evol. Syst.* **46**, 523–549 (2015).

137. Hughes, A. C. *et al.* Sampling biases shape our view of the natural world. *Ecography* **44**, 1259–1269 (2021).

138. Johnson, T. F., Isaac, N. J., Paviolo, A. & González-Suárez, M. Handling missing values in trait data. *Glob. Ecol. Biogeogr.* **30**, 51–62 (2021).

139. Molina-Venegas, R. *et al.* Assessing among-lineage variability in phylogenetic imputation of functional trait datasets. *Ecography* **41**, 1740–1749 (2018).

140. Kattge, J. *et al.* TRY–a global database of plant traits. *Glob. Change Biol.* **17**, 2905–2935 (2011).

141. Myhrvold, N. P. *et al.* An amniote life-history database to perform comparative analyses with birds, mammals, and reptiles. *Ecology* **96**, 3109 (2015).

142. Oliveira, B. F., São-Pedro, V. A., Santos-Barrera, G., Penone, C. & Costa, G. C. AmphiBIO, a global database for amphibian ecological traits. *Sci. Data* **4**, 170123 (2017).

143. Etard, A., Morrill, S. & Newbold, T. Global gaps in trait data for terrestrial vertebrates. *Glob. Ecol. Biogeogr.* **29**, 2143–2158 (2020).

144. Contu, S. *et al.* Release of data added to the PREDICTS database (November 2022). (2022) doi:10.5519/JG7I52DG.

145. Gaston, K. J. & Blackburn, T. M. Birds, body size and the threat of extinction. *Phil. Trans. R. Soc. B* **347**, 205–212 (1995).

146. Keinath, D. A. *et al.* A global analysis of traits predicting species sensitivity to habitat fragmentation. *Glob. Ecol. Biogeogr.* **26**, 115–127 (2017).

147. Staude, I. R., Navarro, L. M. & Pereira, H. M. Range size predicts the risk of local extinction from habitat loss. *Glob. Ecol. Biogeogr.* **29**, 16–25 (2020).

148. Boyles, J. G. & Storm, J. J. The perils of picky eating: dietary breadth is related to extinction risk in insectivorous bats. *PLoS One* **2**, 672 (2007).

149. Kellner, K. F., Duchamp, J. E. & Swihart, R. K. Niche breadth and vertebrate sensitivity to habitat modification: signals from multiple taxa across replicated landscapes. *Biodiv. Conserv.,* **28**, 2647–2667 (2019).

150. Claramunt, S., Hong, M. & Bravo, A. The effect of flight efficiency on gap-crossing ability in Amazonian forest birds. *Biotropica* **54**, 860–868 (2022).

151. Rochat, E., Manel, S., Deschamps-Cottin, M., Widmer, I. & Joost, S. Persistence of butterfly populations in fragmented habitats along urban density gradients: Motility helps. *Heredity* **119**, 328–338 (2017).

152. Etard, A. & Newbold, T. Species-level correlates of land-use responses and climate-change sensitivity in terrestrial vertebrates. *Conserv. Biol.* **38**, 14208 (2024).

153. Maron, J. L., Horvitz, C. C. & Williams, J. L. Using experiments, demography and population models to estimate interaction strength based on transient and asymptotic dynamics. *J. Ecol.* **98**, 290–301 (2010).

154. Williams, J. J., Bates, A. E. & Newbold, T. Human-dominated land uses favour species affiliated with more extreme climates, especially in the tropics. *Ecography* **43**, 391–405 (2020).

155. Freeman, B. G., Scholer, M. N., Ruiz-Gutierrez, V. & Fitzpatrick, J. W. Climate change causes upslope shifts and mountaintop extirpations in a tropical bird community. *Proc. Natl. Acad. Sci. USA* **115**, 11982–11987 (2018).

156. Urban, M. C. Escalator to extinction. *Proc. Natl. Acad. Sci. USA* **115**, 11871–11873 (2018).

157. White, R. L. & Bennett, P. M. Elevational distribution and extinction risk in birds. *PLoS One* **10**, 0121849 (2015).

158. Einoder, L. D. & Richardson, A. M. Aspects of the hindlimb morphology of some Australian birds of prey: a comparative and quantitative study. *Auk* **124**, 773–788 (2007).

159. Miles, D. B. & Ricklefs, R. E. The Correlation Between Ecology and Morphology in Deciduous Forest Passerine Birds. *Ecology* **65**, 1629–1640 (1984).

160. Miles, D. B., Ricklefs, R. E. & Travis, J. Concordance of ecomorphological relationships in three assemblages of passerine birds. *Am. Nat.* **129**, 347–364 (1987).

161. Hudson, L. N., Isaac, N. J. & Reuman, D. C. The relationship between body mass and field metabolic rate among individual birds and mammals. *J. Anim. Ecol.* **82**, 1009-1020 (2013).

162. Steuer, P., Hummel, J., Grosse-Brinkhaus, C. & Südekum, K. H. Food intake rates of herbivorous mammals and birds and the influence of body mass. *Eur. J. Wildl. Res.* **61**, 91–102 (2015).

163. Weeks, B. C. *et al.* Morphological adaptations linked to flight efficiency and aerial lifestyle determine natal dispersal distance in birds. *Funct. Ecol.* **36**, 1681–1689 (2022).

164. González-Varo, J. P. *et al.* Frugivore-mediated seed dispersal in fragmented landscapes: Compositional and functional turnover from forest to matrix. *Proc. Natl. Acad. Sci. USA* **120**, 2302440120 (2023).
