# Supplementary material for: Land-use change undermines the stability of avian functional diversity
Source: Nature. 2025 Nov 26;649(8096):381–7. doi: 10.1038/s41586-025-09788-0 (PMC12779574; doi:10.1038/s41586-025-09788-0)
Supplement: Supplementary file 2 — Reporting Summary [file 41586_2025_9788_MOESM2_ESM.pdf]

nature portfolio

Corresponding author(s): Joe Tobias, Thomas Weeks

Last updated by author(s): Sep 18, 2025

Reporting Summary

Nature Portfolio wishes to improve the reproducibility of the work that we publish. This form provides structure for consistency and transparency in reporting. For further information on Nature Portfolio policies, see our Editorial Policies and the Editorial Policy Checklist.

Statistics

For all statistical analyses, confirm that the following items are present in the figure legend, table legend, main text, or Methods section.

n/a	Confirmed	
☐	☒	The exact sample size (*n*) for each experimental group/condition, given as a discrete number and unit of measurement
☒	☐	A statement on whether measurements were taken from distinct samples or whether the same sample was measured repeatedly
☐	☒	The statistical test(s) used AND whether they are one- or two-sided<br>*Only common tests should be described solely by name; describe more complex techniques in the Methods section.*
☐	☒	A description of all covariates tested
☐	☒	A description of any assumptions or corrections, such as tests of normality and adjustment for multiple comparisons
☐	☒	A full description of the statistical parameters including central tendency (e.g. means) or other basic estimates (e.g. regression coefficient) AND variation (e.g. standard deviation) or associated estimates of uncertainty (e.g. confidence intervals)
☐	☒	For null hypothesis testing, the test statistic (e.g. *F*, *t*, *r*) with confidence intervals, effect sizes, degrees of freedom and *P* value noted<br>*Give P values as exact values whenever suitable.*
☒	☐	For Bayesian analysis, information on the choice of priors and Markov chain Monte Carlo settings
☐	☒	For hierarchical and complex designs, identification of the appropriate level for tests and full reporting of outcomes
☐	☒	Estimates of effect sizes (e.g. Cohen's *d*, Pearson's *r*), indicating how they were calculated

Our web collection on statistics for biologists contains articles on many of the points above.

Software and code

Policy information about availability of computer code

Data collection	No code was used for data collection. Data was collected by contacting reasearchers for their survey data. Data wrangling of AVONET and PREDICTS datasets used tidyverse (Version 2.0.0) in R (Version 4.4.2). All code is available at: 10.5281/zenodo.17184411
Data analysis	Data analysis was undertaken using R (Version 4.4.2). We used the TPD package (version 1.1.0) to calculate functional diversity, redundancy and vulnerability metrics. MESS (Version 0.5.12) to calculate SUC resistance metric. We used lme4 (Version 1,1-36) for linear mixed effects models. Gawdis (Version 0.1.5) to generate distance matrices. Ks (Version 1.15.1) for plug-in density kernels. No custom codes were used for analysis. All code is available at: 10.5281/zenodo.17184411

For manuscripts utilizing custom algorithms or software that are central to the research but not yet described in published literature, software must be made available to editors and reviewers. We strongly encourage code deposition in a community repository (e.g. GitHub). See the Nature Portfolio guidelines for submitting code & software for further information.

Data

Policy information about availability of data

All manuscripts must include a data availability statement. This statement should provide the following information, where applicable:

- Accession codes, unique identifiers, or web links for publicly available datasets
- A description of any restrictions on data availability
- For clinical datasets or third party data, please ensure that the statement adheres to our policy

All data are available at 10.5281/zenodo.17184411. Original survey datasets are available from PREDICTS (https://doi.org/10.5519/jg7i52dg). Bird trait database is available from AVONET (https://figshare.com/s/b990722d72a26b5bfead).

Research involving human participants, their data, or biological material

Policy information about studies with human participants or human data. See also policy information about sex, gender (identity/presentation), and sexual orientation and race, ethnicity and racism.

Reporting on sex and gender	NA
Reporting on race, ethnicity, or other socially relevant groupings	NA
Population characteristics	NA
Recruitment	NA
Ethics oversight	NA

Note that full information on the approval of the study protocol must also be provided in the manuscript.

Field-specific reporting

Please select the one below that is the best fit for your research. If you are not sure, read the appropriate sections before making your selection.

☐ Life sciences ☐ Behavioural & social sciences ☒ Ecological, evolutionary & environmental sciences

For a reference copy of the document with all sections, see nature.com/documents/nr-reporting-summary-flat.pdf

Ecological, evolutionary & environmental sciences study design

All studies must disclose on these points even when the disclosure is negative.

Study description	We calculate various functional metrics for 1281 bird assemblages globally. We test how land-use change effects the functional diversity, functional redundancy, functional vulnerability & ultimately functional resistance using hierarchical mixed effects linear models. We compare the functional metrics in least disturbed habitats to those in disturbed/human-modified habitats.
Research sample	1281 bird assemblages. This was selected to generate a global sample. The majority of data is extracted from the PREDICTS database (https://data.nhm.ac.uk/dataset/release-of-data-added-to-the-predicts-database-november-2022 & https://data.nhm.ac.uk/dataset/the-2016-release-of-the-predicts-database) . We also add 2 studies from the Amazon (https://doi.org/10.1073/pnas.2202310119) and Bornean rainforests (https://doi.org/10.1890/14-0010.1) to ensure we represent the most undisturbed areas.
Sampling strategy	Selection from the PREDICTS database to ensure consistency amongst studies. We removed various unacceptable studies which did not target the entire assemblage or contain abundance information.
Data collection	All data is recorded by on-the-ground samplers from 98 different published studies. Details can be found in the supplementary data. All have been accessed from the following 4 sources: PREDICTS database (https://data.nhm.ac.uk/dataset/release-of-data-added-to-the-predicts-database-november-2022 & https://data.nhm.ac.uk/dataset/the-2016-release-of-the-predicts-database). We also add 2 studies from the Amazon (https://doi.org/10.1073/pnas.2202310119) and Bornean rainforests (https://doi.org/10.1890/14-0010.1)
Timing and spatial scale	We selected datasets from 1990-present. This was to be in keeping with the land use harmonization project which can be used to project our data into the future. This also allowed us to be in keeping with other studies from the same dataset. Spatial scale was global sets of assemblages, from different published studies. Each study varied in geographical extent ranging from less than 1km² to 125,000km²
Data exclusions	Exclusion criteria was based on whether or not the sampling design targetted the entire assemblage and whether the data was published with abundances rather than presence-absence.

Reproducibility	All attempts to repeat analyses were successful
Randomization	Species were grouped into assemblages. Assemblages were based upon predefined studies and study-blocks. A single study-block can contain multiuple assemblages, but all assemblages surveyed within same study block were surveyed in the sampling season and are in close proximity. We used hierarchical modelling with study and study block used as random effects to ensure assemblages are effectively compared within study blocks.
Blinding	Blinding was not used as data had already been acquired by the PREDICTS project. To maximize sample size we selected all appropriate studies from the PREDICTS database. This allows us a global sample.

Did the study involve field work? ☐ Yes ☒ No

Reporting for specific materials, systems and methods

We require information from authors about some types of materials, experimental systems and methods used in many studies. Here, indicate whether each material, system or method listed is relevant to your study. If you are not sure if a list item applies to your research, read the appropriate section before selecting a response.

Materials & experimental systems

n/a	Involved in the study
☒	☐ Antibodies
☒	☐ Eukaryotic cell lines
☒	☐ Palaeontology and archaeology
☒	☐ Animals and other organisms
☒	☐ Clinical data
☒	☐ Dual use research of concern
☒	☐ Plants

Methods

n/a	Involved in the study
☒	☐ ChIP-seq
☒	☐ Flow cytometry
☒	☐ MRI-based neuroimaging

Plants

Seed stocks	NA
Novel plant genotypes	NA
Authentication	NA
